# Are Trace Elements Provided for Children on Long-Term Parenteral Nutrition Adequate to Meet Their Needs?

**DOI:** 10.3390/life15010029

**Published:** 2024-12-30

**Authors:** Hanna Romanowska, Mikołaj Danko, Anna Borkowska, Katarzyna Popińska, Marta Sibilska, Joanna Żydak, Joanna Wielopolska, Klaudia Bartoszewicz, Agnieszka Szlagatys-Sidorkiewicz, Janusz Książyk

**Affiliations:** 1 Department of Pediatrics, Endocrinology, Diabetology, Metabolic Diseases and Cardiology of the Developmental Age, Pomeranian Medical University, 71-252 Szczecin, Poland; joannakastrau89@gmail.com (J.W.); klaudia.bartoszewicz@gmail.com (K.B.); 2 The Children’s Memorial Health Institute, Department of Pediatrics, Nutrition and Metabolic Diseases, 04-730 Warsaw, Poland; m.danko@ipczd.pl (M.D.); kasia.popinska@wp.pl (K.P.); msibilska@poczta.onet.pl (M.S.); janusz@ksiazyk.pl (J.K.); 3 Department of Pediatrics, Gastroenterology, Allergology and Nutrition, Medical University of Gdańsk, 80-803 Gdańsk, Poland; anna.borkowska@gumed.edu.pl (A.B.); agnieszka.szlagatys-sidorkiewicz@gumed.edu.pl (A.S.-S.)

**Keywords:** children, parenteral nutrition, trace elements, cross-sectional study

## Abstract

**Background**: We conducted a cross-sectional study to investigate whether children receiving long-term parenteral nutrition (LPN) are at risk of imbalances in selected trace elements. **Methods**: Serum levels of manganese, zinc, copper, selenium, and iodine were measured in 83 children on LPN and compared with 121 healthy controls. Children with signs of infection or elevated C-reactive protein levels were excluded. Elemental analysis was performed using inductively coupled plasma mass spectrometry (ICP-MS). **Results**: Manganese and copper levels were significantly lower in the study group compared with controls (*p* < 0.001) but remained within normal ranges. Iodine levels were also significantly lower in the study group (*p* < 0.05), though pediatric reference values are lacking. Zinc and selenium levels were significantly higher in the study group (*p* < 0.001), with median levels within normal ranges in both groups. Zinc, selenium, and iodine levels were higher in patients weighing ≤15 kg (*p* < 0.001, *p* < 0.001, *p* < 0.02). **Conclusions**: Serum concentrations of manganese, copper, and selenium in the study group remained within normal ranges, even though children weighing over 15 kg received doses below those recommended by scientific guidelines. An iodine intake below 1 μg/kg/day in patients weighing over 15 kg appears insufficient. Patients on LPN required a higher zinc intake than current recommendations.

## 1. Introduction

Manganese (Mn), selenium (Se), copper (Cu), zinc (Zn), and iodine (I) are essential trace elements (TEs) that play crucial roles in human health. In patients undergoing parenteral nutrition (PN), they are integral components alongside macronutrients in the nutritional mixture [1,2]. Mn, Se, Cu, and Zn serve as cofactors for numerous enzymes, have antioxidant properties, and significantly contribute to the proper functioning of the immune and nervous systems. Moreover, Cu and Mn are essential for collagen synthesis, hemostasis regulation, and carbohydrate metabolism. I and Se are significant for thyroid hormone synthesis, while Zn acts as a mediator in the endocrine, paracrine, and autocrine systems, and is essential for protein and nucleic acid synthesis [1,3,4,5,6,7,8].

Both deficiency and excess of these TEs can result in severe systemic complications. Manganese deficiency is very rare in humans. The primary concern, however, lies in its toxicity, which primarily manifests as irreversible damage to the central nervous system, detectable through magnetic resonance imaging [9]. Se deficiency is associated with diminished resistance to viral infections [10], impaired cognitive function [11], and an increased risk of myopathies [12], while Se overdose elevates the risk of neurodegenerative diseases [13] and type 2 diabetes [11]. Cu deficiency manifests as severe hematological and neurological complications [14,15]. Excessive Cu exposure leads to liver and kidney failure as well as damage to the brain and heart [16]. Clinical symptoms of Zn deficiency encompass growth and sexual maturation delays, immune system dysfunctions, skin alterations, and impaired wound healing [17,18]. The consequence of chronic Zn toxicity is hypocupremia and pancytopenia [19]. Finally, both chronic deficiency and excess iodine intake lead to goiter and thyroid hypofunction [20,21].

Micronutrients should be provided intravenously to all patients receiving PN from the beginning of nutritional support [1]. The gastrointestinal tract predominantly regulates TE concentration in a healthy body. However, with parenteral administration circumventing the gastrointestinal barrier, there is an elevated risk of TE overdose and ensuing toxic symptoms [6,22,23,24]. Children reliant on parenteral nutrition, necessitating the intravenous delivery of all nutritional components, face greater susceptibility to both TE deficiency and excess compared to orally fed healthy children [4,22,23,25,26,27,28,29,30].

International scientific societies have established guidelines concerning micronutrient intake (Table 1) [1,2,3,31]. However, there are differences between scientific societies’ recommendations and the dosages manufacturers recommend for commercially available products used in parenteral nutrition. The gastrointestinal tract predominantly regulates TE concentration in a healthy body [6]. There is a lack of studies in the literature examining the TE status in large cohorts of children undergoing long-term parenteral nutrition (LPN). Consequently, our study aimed to answer the question: Are children requiring LPN at risk of imbalances in selected TEs? To this end, we examined the concentrations of Mn, Zn, Cu, Se, and I in the serum of pediatric patients receiving LPN and compared them with the concentrations of these elements in the serum of healthy children serving as a control group.

This study builds upon prior research on the same patient cohort where we investigated aluminum, arsenic, and cobalt exposure in children undergoing LPN [32,33]. It should be emphasized that aluminum constitutes a contaminant in the mixtures used in PN.

## 2. Methods

### 2.1. Study Participants

The study group has been previously described in published articles that delved into aluminum, arsenic, and cobalt exposure within the same cohort of patients [32,33]. As delineated in the preceding articles, the study encompassed patients receiving home parenteral nutrition (HPN) from two medical centers in Poland. We conducted a cross-sectional study involving a cohort of 83 children (31 females and 52 males) from various regions of Poland, aged between 7.2 months and 18 years, who underwent long-term parenteral nutrition (LPN) between 2004 and 2022. The duration of nutritional intervention ranged from 4.6 months to 16.75 years. Indications for HPN included the following: congenital mesenteric torsion (11 patients), Hirschsprung’s disease (14 patients), microvessel inclusion disease (1 patient), intestinal atresia (4 patients), expectoration (7 patients), torsion of the small intestine (5 patients), Berdon syndrome (2 patients), Pagoda syndrome (4 patients), neurogenic bowel (6 patients), absorption disorders (4 patients), uncompleted intestinal diversion (3 patients), necrotizing enterocolitis (12 patients), and ten patients with other congenital intestinal anomalies.

All children received custom nutritional mixtures tailored to their specific requirements, providing over 50% of their total energy intake. Energy intake was calculated individually for each patient in line with ESPGHAN recommendations [34].

Commencing from the initiation of nutritional support, all participants received the intravenous Multi-Trace Element product, Peditrace^®^ (Fresenius Kabi, Wroclaw, Poland), with dosages according to the product’s specifications. The study group was divided into patients with a body weight ≤ 15 kg (BW ≤ 15 kg), consisting of 27 individuals, and patients with a body weight > 15 kg (BW > 15 kg), consisting of 56 individuals. This division of the study group was due to the dosage of the Peditrace^®^ (Fresenius Kabi) preparation, which is dependent on body weight; regardless of the patient’s age, it is 1 mL/kg/day for patients with a body weight up to 15 kg and 15 mL/day for patients with a body weight above 15 kg. Such dosing was consistently administered to patients within our study group, albeit not always aligning with the recommendations of international scientific societies regarding TE provision (Table 1). All patients underwent thorough physical examination, with assessments of body weight conducted. In all study participants, the levels of C-reactive protein, creatinine, and liver function parameters were measured. Patients with elevated levels of C-reactive protein, kidney failure, and cholestasis were excluded from the study cohort. Disinfectants containing iodine were not used on the sampled children. Patients in the study group did not require additional supplementation of trace elements due to gastrointestinal losses.

Due to significant difficulties in establishing reference ranges for certain trace elements in children’s serum, our study results were contextualized against a control group comprising 121 healthy children (54 females, 67 males) meticulously matched with the study cohort regarding age and gender, hailing from northwestern Poland. Each participant was interviewed and physically examined, with body weight and height measurements referenced against WHO percentile charts specific to sex (https://www.ptzkd.org/standardy/ accessed on 22 August 2024). Children with symptoms of infection, elevated levels of C-reactive protein, and abnormal anthropometric parameters were excluded from the control group. Participants in the control group did not receive any dietary supplements during the last six months. All study participants abstained from consuming seafood, fish, and rice for five days prior to blood sample collection. None of the participants in the study nor the control group exhibited clinical symptoms indicative of TE deficiency or excess.

The research adhered to the principles outlined in the Declaration of Helsinki and received approval from the Ethics Committee at the Pomeranian Medical University in Szczecin (reference number KB-0012/21/2020, approval date: 9 March 2020).

### 2.2. The Dosage of Trace Elements in the Study Group and in the Control Group

Table 1 shows the recommendations of the European Society of Pediatric Gastroenterology, Hepatology and Nutrition (ESPGHAN), European Society for Clinical Nutrition and Metabolism (ESPEN), European Society of Pediatric Research (ESPR), Chinese Society of Parenteral and Enteral Nutrition (CSPEN) [3], and American Society for Parenteral and Enteral Nutrition (ASPEN) [31] regarding the intake of TEs in children as well as the supply of TEs in our study group.

The intravenous administration of Mn, Cu, Se, and I in the BW ≤ 15 kg group was in accordance with the recommendations of the scientific societies; in the BW > 15 kg group, it was significantly lower than the recommendations. In contrast, the intravenous administration of Zn in all patients in the study group was significantly higher than the recommendations but did not exceed the recommended maximum dose.

### 2.3. Trace Element Determination

The serum concentrations of Mn, Zn, Cu, Se, and I were meticulously determined for both the study and control cohorts, with sample collection spanning the years 2021 to 2022. Below, we outline the methods employed for identifying TEs in serum, which were detailed in the previously mentioned articles concerning the evaluation of aluminum, arsenic, and cobalt levels within the same study cohort [32,33].

Samples of blood were taken from fasting participants via venipuncture using the Vacutainer^®^ System (BD EST Z #362725, Plymouth, UK). Once collected, the blood was left to clot at room temperature for a period of at least 30 min. The samples were then centrifuged at 1300 g for 12 min to isolate the serum. Following centrifugation, the serum was carefully divided into new cryovials and then frozen at −80 °C until analysis. The elemental composition of the samples was determined using the inductively coupled plasma mass spectrometry (ICP-MS) technique with the NexION 350D instrument (PerkinElmer, Norfolk, VA, USA). The KED (Kinetic Energy Discrimination) mode was employed for element determination, and rhodium was used as an internal standard to compensate for instrument drift and matrix effects. Detailed information regarding the specific parameters of the NexION 350D instrument used in the measurements can be provided upon request. During analysis, the serum samples were diluted 30-fold with a blank reagent.

The blank reagent was composed of ultrapure water (>18 MΩ), TMAH sourced from AlfaAesar (Kandel, Germany), Triton X-100 from PerkinElmer (Shelton, CT, USA), as well as nitric acid and ethyl alcohol, both supplied by Merck (Darmstadt, Germany).

Calibration standards were made by diluting a 10 mg/L Multi-element Calibration Standard 3 solution (PerkinElmer Pure Plus, Shelton, CT, USA) with the blank reagent. A matrix-matching approach was utilized for calibration, ensuring correlation coefficients consistently surpassed 0.999.

### 2.4. Quality Control

To maintain high standards of precision and reliability, the following certified reference materials (CRM) were applied: ClinChek^®^ Plasmonorm Serum Trace Elements Level 1 (Recipe, Munich, Germany) for analyzing serum samples and ClinChek^®^ Urine Control Level 1 (Recipe, Munich, Germany) for evaluating urine samples. Additional specifications regarding plasma operation conditions and mass spectrometer configurations are available upon request. Quality assurance is ensured through active participation in two separate external programs: LAMP (Lead and Multielement Proficiency Program) overseen by the CDC and QMEQAS, administered by the Quebec Public Health Institute.

### 2.5. Statistics

The study group consisted of patients who were receiving long-term parenteral nutrition. A control group of healthy children was selected to match the study group in terms of age and sex. The size of the study group was determined with the assumption that 10% to 15% of the 100 potential participants would not consent to the tests. The confidence level for the study was set at 95%, and the margin of error was established at 5%. As a result, it was necessary to have at least 81 subjects in the study sample.

Statistical analysis was performed using Statistica 7.1 (StatSoft^®^). The Kolmogorov–Smirnov test showed a nonnormal distribution of variables. Continuous data were presented as median, range (min.–max.) and interquartile range (IQR). The Mann–Whitney U test was employed for data analysis. A significance level of *p* < 0.05 was established for determining statistical significance.

## 3. Results

The storage time of samples in both groups did not differ statistically (*p* = 0.52) and ranged from 6 to 22 months in the study group and from 10 to 22 months in the control group.

Table 2 shows the serum TE concentrations in the study and control groups.

Serum Mn, Cu, and I concentrations were significantly higher in the control group, while serum Zn and Se were significantly higher in the study group.

Table 3 shows the serum TE concentrations in the study group divided into groups of patients with BW ≤ 15 kg and patients with BW > 15 kg.

Serum Zn, Se, and I concentrations were significantly higher in the BW ≤ 15 kg group, while Mn and Cu concentrations were not statistically different between patients in the BW ≤ 15 kg and BW > 15 kg groups.

## 4. Discussion

The present study is one of the most extensive studies to determine TE status in children on LPN. The results presented, in line with recommendations from scientific societies, confirm the necessity of TE supplementation in this patient group. However, the dosage of TEs for patients weighing 15 kg may be subject to further discussion.

Most of the available publications indicate the potential complications related to the toxic effects of excessive Mn in children on LPN due to the intravenous administration of this element both in parenteral Multi-Trace Element products and as a contaminant of other products used in PN [4,22,23,25,26]. Based on a US literature review, serum Mn concentrations considered “normal” range from 0.9 to 2.9 μg/L [23,35]. In our study, 25% of the patients in the study group had serum Mn concentrations above the upper range of “normal” reported in the US. No patient was found to be Mn deficient. In the control group, the range of Mn concentrations was surprisingly higher than in the study group, with 86% having concentrations above the upper limit of “normal”. The results of our study in the control group are similar to those of Gaman et al. [36], who found a range of Mn concentrations of 6.7–10.3 μg/L in healthy children The significantly lower Mn concentrations in the study group compared to the control group may indicate a low risk of Mn toxicity in the study group. However, the lack of statistical difference between the BW ≤ 15 kg and BW > 15 kg groups shows that the Mn supply in the BW > 15 kg group was sufficient despite a lower dose than recommended. Because of the asymptomatic hypermanganesemia demonstrated in the study by Siepler et al. [37], magnetic resonance imaging (MRI) of the brain should be performed for an objective assessment of Mn toxicity [26,38,39,40]. Hardy et al. [23] recommend the routine measurement of Mn in whole blood in conjunction with brain NMR to monitor Mn accumulation in patients on LPN

In contrast to manganese (Mn) toxicity, zinc (Zn) toxicity is rare. Zinc deficiencies are more frequently observed in patients with short bowel syndrome, caused by gastrointestinal losses related to factors such as impaired absorption, increased intestinal and gastric secretion, and intestinal fistulas [3,6,41]. An increased intravenous supply of Zn in the study group was confirmed in the conducted analyses, which revealed significantly higher concentrations of Zn in the study group compared to the control group (Table 2). The highest serum Zn concentration was found in the BW ≤ 15 kg group, where the dose per body weight was 2.5–5 times higher than the recommended dose (Table 1 and Table 3). However, the serum Zn concentration median in the study and control groups was within the normal range for Caucasian children in the USA, i.e., 640–1240 μg/L (a study conducted on 2115 healthy children in Utah, USA) [42] (Table 2). Unfortunately, no population studies establish reference ranges for Zn in children in Poland. Patients in the study group who were burdened with intestinal diseases had a higher Zn requirement, consistent with results from Wolman et al. [43] and Btaiche et al. [44].

Both Cu deficiency and toxicity have been described in patients on PN. MacKay et al. [27] demonstrated Cu deficiency in patients receiving PN without TE supplementation. The study by Namjoshi et al. [28] showed that Cu deficiency was one of the most common TE deficiencies in studied patients receiving PN. Excessive Cu accumulation may occur in patients with cholestasis, as shown in a study by Blaszyk et al. [45]. In the study by Lin et al. [42], the reference ranges for Cu varied with age, and the normal range for the study group of 2115 children was 570–1530 μg/L. The serum Cu concentration in our study was significantly lower in the study group than in the control group, but the Cu concentration median in both groups was within the range reported by Lin (Table 2). At the same time, no difference was demonstrated between the BW ≤ 15 kg and BW > 15 kg groups (Table 3). The results indicate that the intravenous Cu supply in our patients was correct, despite being significantly lower than the recommended supply in the BW > 15 kg group (Table 1).

The results of the analysis in our study group do not confirm the Se deficiency described in patients on PN [4,28,29,30]. More than 95% of the children in the study group had serum Se concentrations within the range considered normal in a Korean study (50–150 μg/L) [29], and the median was significantly higher compared with the control group (Table 2). In the BW ≤ 15 kg group, normal serum Se concentrations were found in 100% of patients, which may indicate that a supplementation of 2 μg/kg/d is adequate. However, this is inconsistent with the study by Johnsen et al. [4], where normal serum Se concentrations were only found with a supplementation of 3 μg/kg/d in pediatric patients on PN. Similarly, the study by Lee et al. [29] showed that for neonates and infants, a Se supply greater than 2 μg/kg/d is necessary. The large discrepancy in Se concentrations in the control group (range 25–398 μg/L) indicates the influence of diet.

Intravenous I supplementation in pediatric patients on PN is recommended in Europe [3]. Johnsen et al. [4] described I deficiency, measured in urine, in children on PN without supplementation of this element. In the available literature, we could not find norms for serum I concentration in children. Compared to our control group, we found significantly lower serum I concentrations in the study group (Table 2). The serum I concentration was significantly higher in the BW ≤ 15 kg group compared to the BW > 15 kg group, and the I concentration medians in the control group and the BW ≤ 15 kg group were similar (Table 3). This may suggest that the recommended intravenous I dose of 1 μg/kg/d is sufficient, although it was shown to be suboptimal in a study by Cicalese et al. [46]. An I supply of less than 1 μg/kg/d in the BW > 15 kg group seems to be too low. The monitoring of I concentration is most accurate in urine samples from daily collections, which may be difficult to perform. European recommendations suggest thyroid tests as a replacement marker for assessing I concentration [3].

Several limitations to this study require clarification.

In many published reports, it has been established that measuring Mn in whole blood correlates with the degree of accumulation of this element in the brain and serves as an indicator of Mn toxicity [23,47,48]. This article’s authors know that measuring serum Mn levels is a less accurate method; however, it is commonly used. In addition, the determined concentrations of trace elements (TEs) are compared to the results of studies conducted in distant regions of the world, although, ideally, they should be compared with norms for our region, considering environmental conditions. Unfortunately, no reference ranges exist for Mn, Zn, Cu, Se, and I for children in Poland. Therefore, we primarily compared the results of our study with those of a control group comprised of healthy children from the northwestern region of Poland. We are also aware that the age range of the study group is wide (from 7.2 months to 18 years), but the dosage of the Peditrace^®^ preparation (Fresenius Kabi) is based on body weight and not on age. Thus, we present a group of patients who received the recommended dosage to assess whether it was appropriate and met the needs of the patients in our study group.

## 5. Conclusions

1. The serum concentrations of Mn, Cu, and Se in the study group aligned with the established norms, even with lower-than-recommended doses for patients over 15 kg;
2. An iodine intake below 1 μg/kg/day appears insufficient for patients weighing more than 15 kg;
3. Patients on LPN were shown to require a higher Zn supply than current recommendations;
4. The lower serum Mn levels in the study group compared to the controls suggest a reduced risk of Mn toxicity in these patients;
5. Comprehensive population studies in Poland are needed to define reference ranges for Mn, Zn, Cu, Se, and I across age groups, improving the monitoring of patients on parenteral nutrition.

## Figures and Tables

**Table 1:** Comparison of daily supply of selected TEs in the study group with recommendations of ESPGHAN, ESPEN, ESPR, CSPEN [3], and ASPEN [31].

TE	ESPGHAN, ESPEN, ESPR, CSPEN Recommendations (2018)		A.S.P.E.N Recommendations (2012)		TE Supply in the Study Group	
	Infants Born on Time μg/kg/day	Children > 12 mo μg/kg/day	Infants Born on Time μg/kg/day	Children > 12 mo μg/kg/day	BW ≤ 15 kg μg/kg/day	BW > 15 kg μg/day
Mn (manganese)	1 (max 50 μg/day)	1 (max 50 μg/day)	1 (max 50 μg/day)	1 (max 50 μg/day)	1	15
Zn (zinc)	>3 mo: 100 (no max stated)	50 (max 5000 μg/day)	>3 mo: 50 (max 5000 μg/day)	50 (max 5000 μg/day)	250	3750
Cu (copper)	20 (no max stated)	20 (max 500 μg/day)	20 (no max stated)	20 (max 500 μg/day)	20	300
Se (selenium)	2–3 (no max stated)	2–3 (max 100 μg/day)	1–3 (no max stated)	1–3 (max 100 μg/day)	2	30
I (iodine)	At least 1 (no max stated)	At least 1 (no max stated)	1 (no max stated)	1 (no max stated)	1	15

BW—body weight.

**Table 2:** Comparison of serum TE concentrations in the study and control groups expressed in μg/L.

Trace Elements	Study Group			Control Group			*p*-Value
	Median (μg/L)	Range (Min–Max) (μg/L)	Interquartile Range (IQR)	Median (μg/L)	Range (Min–Max) (μg/L)	Interquartile Range (IQR)	
Mn (manganese)	2.42	0.91–7.27	1.0	3.95	1.9–63.55	1.58	*p* < 0.001
Zn (zinc)	985.68	449.16–1781.95	354.3	774.51	480.0–1955.36	210.58	*p* < 0.001
Cu (copper)	915.99	283.58–1876.53	167.4	1071.0	327.09–2318.6	354.97	*p* < 0.001
Se (selenium)	75.53	41.08–118.52	21.36	61.18	25.49–397.79	13.58	*p* < 0.001
I (iodine)	61.75	32.1–348.07	16.55	65.8	44.89–129.56	17.02	*p* < 0.05

**Table 3:** Comparison of serum TE concentrations expressed in μg/L in the study group, divided into BW ≤ 15 kg and BW > 15 kg groups.

Trace Elements	BW ≤ 15 kg			BW > 15 kg			*p*-Value
	Median (μg/L)	Range (Min–Max) (μg/L)	Interquartile Range (IQR)	Median (μg/L)	Range (Min–Max) (μg/L)	Interquartile Range (IQR)	
Mn (manganese)	2.55	1.2–7.27	1.27	2.32	0.91–6.19	0.96	NS
Zn (zinc)	1107.71	710.41–1781.95	211.8	896.75	449.16–1664.55	333.34	*p* < 0.001
Cu (copper)	949.23	651.08–1876.56	149.75	885.37	283.58–1392.28	226.5	NS
Se (selenium)	84.68	52.33–118.52	13.17	71.15	41.08–105.89	18.62	*p* < 0.001
I (iodine)	65.84	44.35–384.07	19.76	59.33	32.1–102.72	14.6	*p* < 0.02

BW—body weight; NS—statistically nonsignificant (*p* > 0.05).

## Data Availability

The original contributions presented in this study are included in the article/Supplementary Material. Further inquiries can be directed to the corresponding author.

## Supplementary Materials

The following supporting information can be downloaded at: https://www.mdpi.com/article/10.3390/life15010029/s1, Table S1: Database: Trace elements—study group and control group.
